# NEAT1/miR-200b-3p/SMAD2 axis promotes progression of melanoma

**DOI:** 10.18632/aging.103909

**Published:** 2020-11-16

**Authors:** Wen-Jie Zhou, Hao-Yu Wang, Jie Zhang, Hai-Ying Dai, Zhi-Xian Yao, Zhong Zheng, Sun Meng-Yan, Ke Wu

**Affiliations:** 1 Department of Gynecology and Obstetrics, Reproductive Medical Center, Shanghai Ruijin Hospital, Shanghai Jiao Tong University School of Medicine, Shanghai 200025, China; 2 Department of Dermatology, Shanghai Ninth People’s Hospital, Shanghai Jiao Tong University School of Medicine, Shanghai 200025, China; 3 Department of Obstetrics and Gynecology, Shanghai General Hospital, Shanghai Jiao Tong University School of Medicine, Shanghai 200080, China; 4 Department of Plastic Surgery, Changhai Hospital, Naval Medical University, Shanghai 200433, China; 5 Department of Urology, Shanghai General Hospital, Shanghai Jiao Tong University School of Medicine, Shanghai 200080, China

**Keywords:** miR-200b-3p, SMAD2, NEAT1, melanoma, EMT

## Abstract

Melanoma is a skin malignancy with a high mutation frequency of genetic alterations. MicroRNA (miR)-200b-3p is involved in various cancers, while in melanoma its bio-function remains unknown. In this study, we found that miR-200b-3p was down-regulated in melanoma tissues and cell lines compared to benign nevus cells. Overexpression of miR-200b-3p significantly inhibited the proliferation and invasion of melanoma cells. According to bioinformatics analysis and sequencing data, we supposed that SMAD family member 2 (SMAD2) was the target gene and nuclear enriched abundant transcript 1 (NEAT1) was the upstream long non-coding RNA (lncRNA) of miR-200b-3p. These predictions were verified by western blotting and quantitative real-time reverse transcription PCR (RT-qPCR). Luciferase reporter assays revealed that NEAT1 up-regulated SMAD2 by directly sponging miR-200b-3p. *In vitro* and *in vivo*, we demonstrated that both NEAT1 and SMAD2 could promote the proliferation and invasion of melanoma cells, and these effects were reversed by up-regulating miR-200b-3p. In addition, NEAT1/miR-200b-3p/SMAD2 axis promoted melanoma progression by activating EMT signaling pathway and immune responses. Taken together, the NEAT1/miR-200b-3p/SMAD2 signaling pathway promotes melanoma via activation of EMT, cell invasion and is related with immune responses, which provides new insights into the molecular mechanisms and therapeutic targets for melanoma.

## INTRODUCTION

Melanoma is one of the most malignant skin cancers with an increasing incidence [1], and accounts for approximately 80% skin-cancer related deaths in the worldwide [2]. Genetic landscape of malignancies demonstrates that melanoma involves the highest mutation frequency of genetic alterations among all cancers analyzed [3]. The prognosis of metastatic melanoma remains unsatisfactory despite the application of standard as well as novel therapies [4]. Therefore, it is essential to identify potential genetic biomarkers thus providing new insight into the mechanism of tumorigenesis and progression of melanoma.

miRNAs, which are characterized as small, single-stranded noncoding RNAs (ncRNAs), can target specific mRNAs to inhibit protein translation. miR-200b-3p is an important member of the miR-200 family, which is associated with epithelial-to-mesenchymal transition (EMT) and mesenchymal-to-epithelial transition (MET), cancer cell proliferation, and drug resistance [5, 6]. It has been reported that miR-200b-3p plays a part in the regulation of several cancer [7–11]. Nonetheless, little is known about the roles of miR-200b-3p in melanoma progression, especially its signaling pathway, crosstalk with some lncRNAs and proteins in the tumorigenesis.

SMAD family member 2 (SMAD2) is a member of the receptor-regulated SMADs and serves as an intracellular signal transducer and transcriptional modulator downstream the TGF-β signaling pathway. It has been reported that SMAD2 is invloved in tissue differentiation, fetal development, inflammatory responses, and even in tumorigenesis. Altered status of SMAD2 may lead to an interrupt signaling of TGF-β, thus tumor cells can escape the growth inhibiting effect of TGF-β [12]. Previous research has established that SMAD2 is a target gene of some specific miRNAs in other tumors. For example, SMAD2 promoted pancreatic cancer progression via interacting with miR-655 [13]. For lung cancer, miR-433 and miR-27a suppresses tumor progression via targeting SMAD2 [14, 15]. Interestingly, these functional miRNAs are also found to be sponged by their corresponding lncRNAs, thus forming a regulatory axis of lncRNA/miRNA/mRNA in the progression, migration, and invasion of cancers.

Among numerous tumor-related lncRNAs, whose length ranges from 200nt to 100kb without capacity of protein-coding function [16], nuclear enriched abundant transcript 1 (NEAT1) is a novel lncRNA localized specifically to nuclear paraspeckles [17] and transcribed from multiple endocrine neoplasia type 1 locus [18]. The dys-regulation and malfunction of NEAT1 have been related to various cancers, including breast cancer, colorectal cancer, ovarian carcer, cervical cancer, lung cancer, etc [19–22]. However, the relationship between miR-200b-3p and NEAT1 in melanoma has not been fully elucidated.

Considering the correlations among miR-200b-3p, SMAD2 and NEAT1, the aim of this study is to investigate biofunction of NEAT1/miR-200-3p/SMAD2 axis in melanoma and explore its potential as a novel biomarker and therapeutic target of melanoma.

## RESULTS

### miR-200b-3p was down-regulated in malignant melanoma and associated with poor overall survival

**Figure 1:**
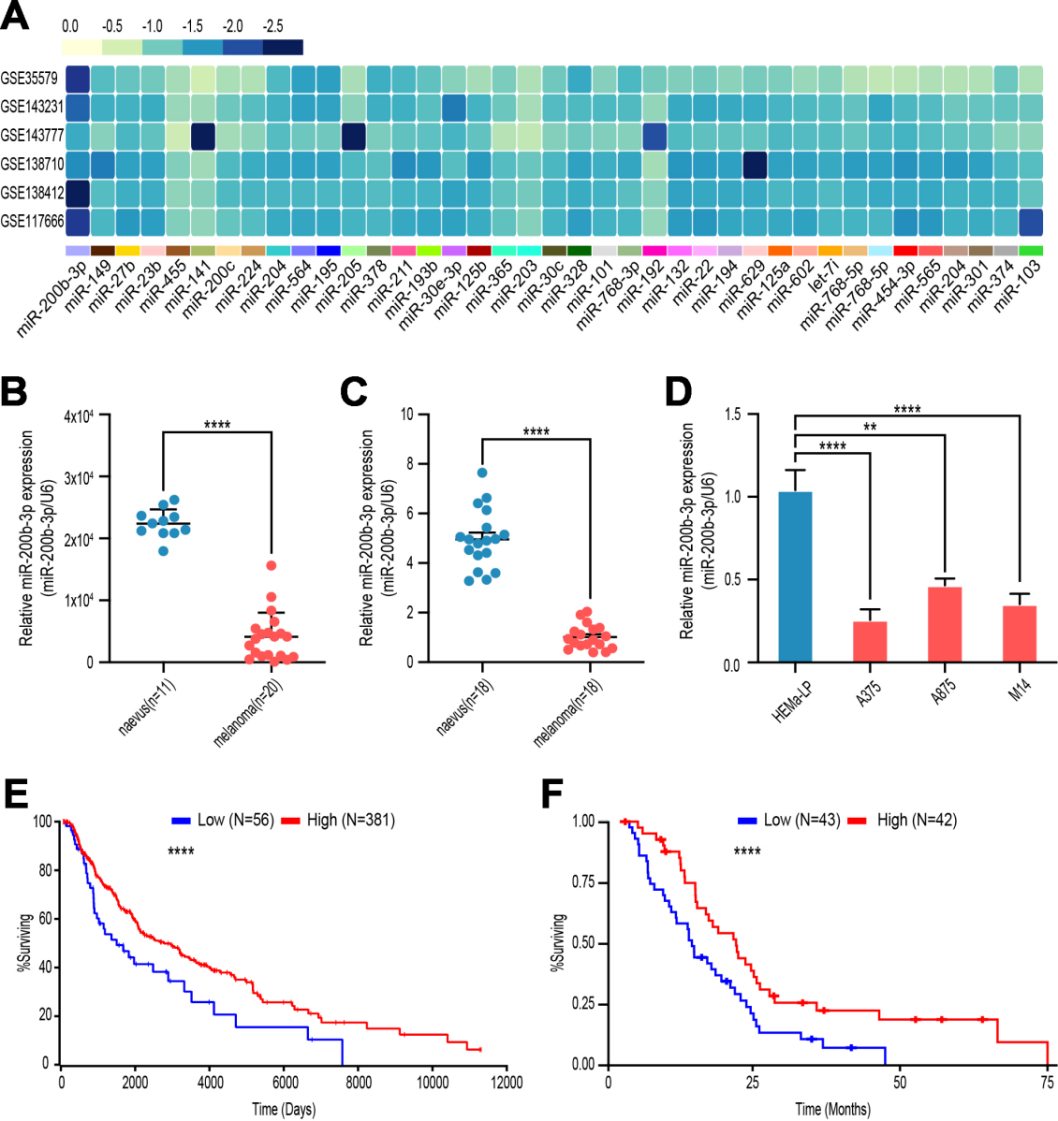
**miR-200b-3p was down-regulated in malignant melanoma and associated with poor overall survival.** (**A**) GSE35579, GSE143231, GSE143777, GSE138710, GSE138412, GSE117666 profiles were analyzed to screen melanoma-related miRNAs. (**B**) Levels of miR-200b-3p between benign nevus (n=11) and malignant melanoma (n=20) tissues were analyzed according to GSE35579. (**C**) Levels of miR-200b-3p of clinic samples between benign nevus (n=18) and malignant melanoma tissues (n=18) were measured by RT-qPCR. (**D**) Levels of miR-200b-3p in melanoma cells lines (A375, A875 and M14) compared to benign epithelial cell, HEMa-LP, by RT-qPCR. (**E**) Kaplan–Meier survival curves for melanoma patients with high (n=56) or low (n=381) miR-200b-3p levels. (**F**) Kaplan–Meier survival curves for mesothelioma patients with high (n=43) or low (n=42) miR-200b-3p levels.

To explore the potential aberrant miRNAs in melanoma, we analyzed gene microarray from GEO database (GSE35579, GSE143231, GSE143777, GSE138710, GSE138412and GSE117666), and found that miR-200b-3p was the most down-regulated among the top down-regulated miRNAs (Figure 1A). Next, level of miR-200b-3p in malignant melanoma tissues was lower than that in benign nevus according to GSE35579 (Figure 1B). Moreover, verified by our own clinic samples and cell lines, expression of miR-200b-3p was decreased in melanoma tissues (n=18) compared with benign nevus (n=18) (Figure 1C). It is similar that miR-200b-3p significantly decreased in melanoma cell lines (A375, A875 and M14) compared with benign epithelial cell line (HEMa-LP) (Figure 1D). To determine whether miR-200b-3p could serve as a prognostic biomarker, Kaplan-Meier analysis was performed according to TCGA database. The results indicated that lower expression of miR-200b-3p in patients with melanoma was associated with poor prognosis (Figure 1E). To further confirm the results above, we analyzed the overall survival time of mesothelioma patients, who shared similar pathological characteristics with melanoma patients. The Kaplan-Meier analysis showed that patients with high miR-200b-3p expression tend to live longer (Figure 1F). These consistent results suggested that miR-200b-3p might act as a prognostic biomarker for several cutaneous malignant tumor.

### miR-200b-3p inhibited proliferation and invasion of melanoma cells

**Figure 2:**
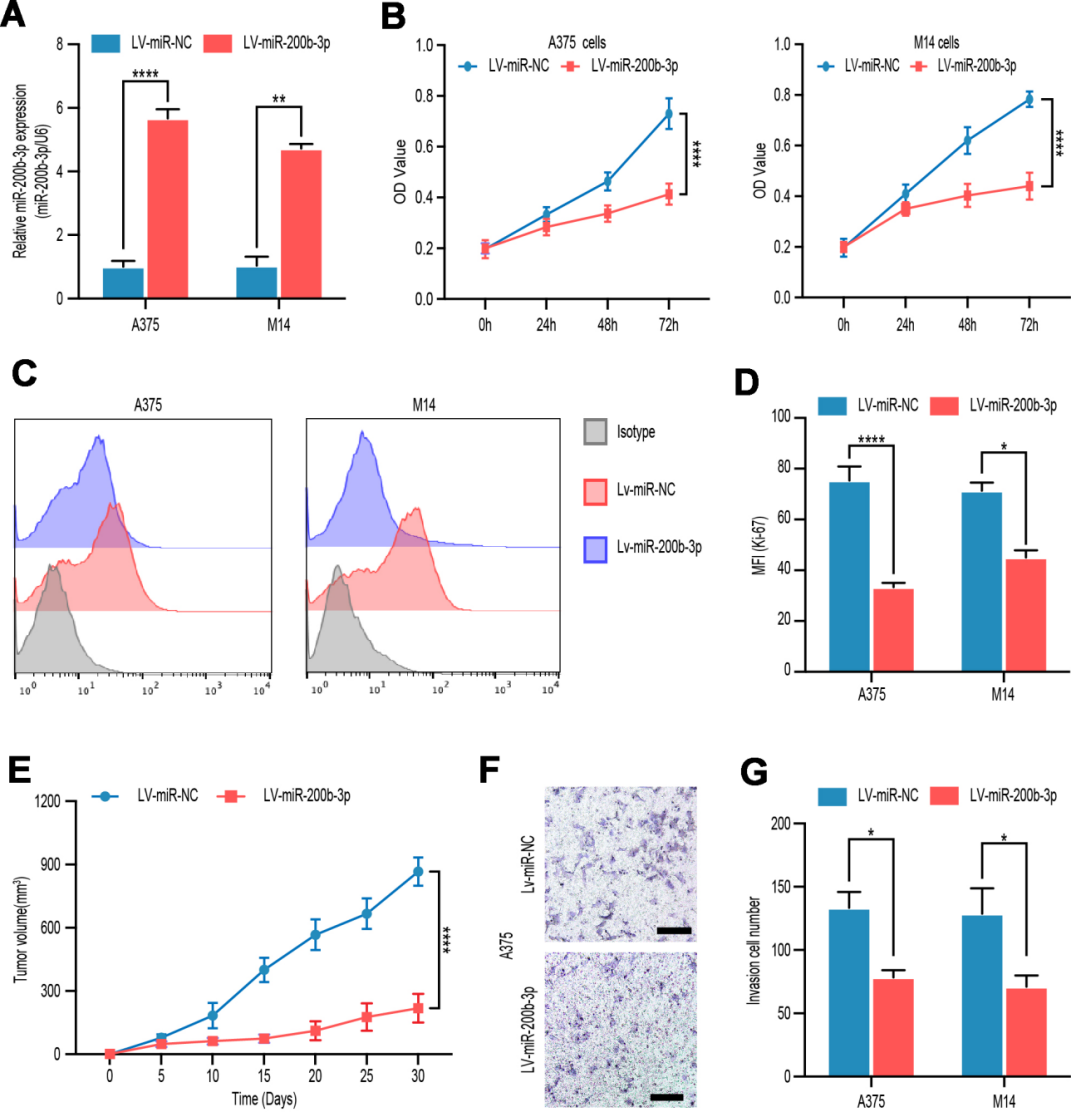
**miR-200b-3p inhibited proliferation and invasion of melanoma cells.** (**A**) A375 and M14 were transfected with LV-miR-NC or LV-miR-200b-3p for 24 hours and RT-qPCR was used to access miR-200b-3p levels. (**B**) CCK-8 assays were used to identify cell proliferation of LV-miR-200b-3p-transfected melanoma cells compared with that of control cells. (**C**–**D**) Following treatment for 48 hours, Ki67 was tested by flow cytometry in LV-miR-200b-3p-transfected cell lines compared with that of control cells. (**E**) Tumor growth curves were calculated after A375 cells transfected with miR-200b-3p. (**F**–**G**) Cell invasion was detected after cells transfected with LV-miR-200b-3p or control at 24h. Scale bars: 100 μm.

To explore the function of miR-200p-3b in melanoma cells, we overexpressed miR-200p-3b in A375 and M14 cells by lentiviral transfection and a high-efficiency of interference was verified by RT-qPCR (Figure 2A). Compared with the miR-control groups, transduction with LV-miR-200p-3b inhibited proliferation in A375 and M14 cell lines according to CCK-8 assay (Figure 2B). Ki-67 is considered a good marker of proliferation. At least 10000 cells were detected in flow cytometry assays and statistical data showed that miR-200p-3b overexpression inhibited cell proliferation proved by a decreased level of Ki-67 (Figure 2C, 2D). *In vivo*, a xenograft mouse model was constructed after LV-miR-200b-3p or mock transfected A375 cells were subcutaneously injected in nude mice. Subsequently, we closely observed the development of tumors and noticed that miR-200b-3p overexpression xenografts developed much slower than those of the mock group (Figure 2E). As for invasion, transwell assays showed that overexpression of miR-200b-3p reduced invasion of melanoma cells (Figure 2F, 2G). These results revealed that miR-200b-3p inhibited cell proliferation and invasion in melanoma cells.

### SMAD2 was verified as a functional target of miR-200b-3p

**Figure 3:**
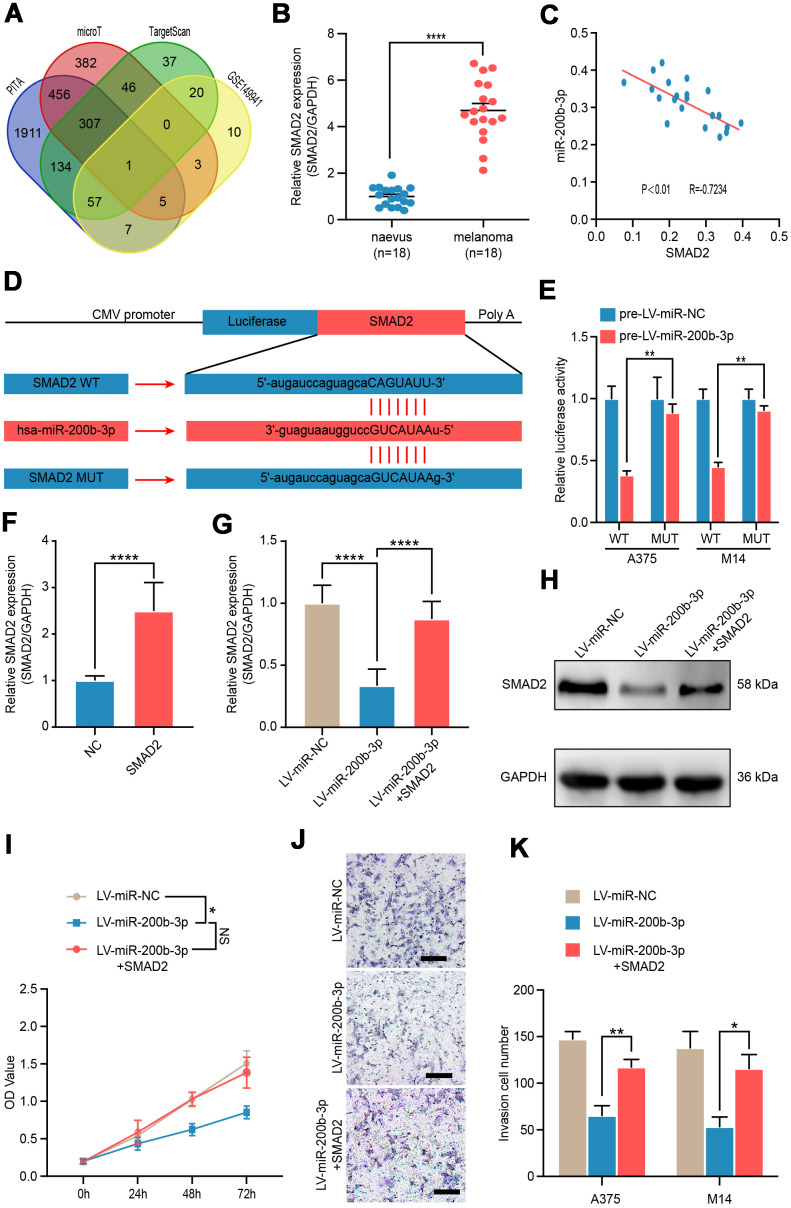
**SMAD2 was verified as a functional target of miR-200b-3p.** (**A**) Venn diagram of intersection of miRNA-200b-3p target genes predicted by bioinformatics analysis. (**B**) RT-qPCR was used to test expression of SMAD2 in benign nevus (n=18) and malignant melanoma tissues (n=18). (**C**) Spearman’s correlation analysis showed the correlation of miR-200b-3p and SMAD2 in malignant melanoma tissues (n=18). (**D**) Schematic view of putative miRNA-200b-3p targeting site in the Wt and Mut 3’-untranslated region (UTR) of SMAD2. (**E**) Luciferase reporter assay in A375 and M14 cells transfected with luciferase report plasmids containing SMAD2 3’- UTR (WT or MUT), and control miRNA or LV-miRNA-200b-3p. (**F**) RT-qPCR was used to test the efficiency of SMAD2 overexpression plasmid. (**G**–**H**) RT-qPCR and western blot were used to evaluate the mRNA and protein levels of SMAD2, after LV-miR-200b-3p and/or SMAD2 up-regulated lentivirus respectively. (**I**) CCK-8 assays were conducted to identify cell proliferation after cells were transfected LV-miR-NC, LV-miR-200b-3p, or LV-miR-200b-3p+SMAD2. (**J**–**K**) Cell invasion was detected after cells were transfected with LV-miR-NC, LV-miR-200b-3p, or LV-miR-200b-3p+SMAD2 or control at 24h. Scale bars: 100 μm.

To identify the possible target gene of miR-200b-3p in melanoma, bioinformatics analysis was conducted using several bioinformatics databases including PITA, microT, TargetScan and RNA sequencing data of GSE149941. SMAD2 was the only one potential target gene in the interaction (Figure 3A). Hence, we verified the expression of SMAD2 in benign nevus (n=18) and malignant melanoma tissues (n=18) by RT-qPCR. It was shown that SMAD2 was significantly upregulated in melanoma tissues compared with benign naevus (Figure 3B). In addition, Spearman’s correlation analysis depicted a negative correlation between expressions of miR-200b-3p and SMAD2 in the 18 melanoma tissue samples (R=−0.7234, P < 0.01) (Figure 3C). Multiple sequence comparison analysis indicated that there was a possible miR-200b-3p binding site in the SMAD2 3'-UTR sequence (Figure 3D). Thus, in A375 and M14 cells, luciferase reporter assays were conducted to identify whether SMAD2 was directly bound by miR-200b-3p. We constructed vectors encoding the partial 3'-UTR sequence of SMAD2, which included the potential miR-200b-3p binding site. Following co-transduction with LV-miR-200b-3p and the vector carrying the wild-type sequence containing the SMAD2 3'-UTR, the luminescence intensity of SMAD2 was significantly reduced, which implied a direct binding of miR-200b-3p and SMAD2 (Figure 3E). Next, we constructed SMAD2 overexpression plasmid and the efficiency was confirmed by RT-qPCR (Figure 3F). Rescue assays showed that LV-miRNA-200b-3p effectively down-regulated SMAD2, while SMAD2 overexpression plasmid could reverse this phenomenon in both of genetic and protein levels (Figure 3G, 3H). As for bio-function, up-regulated SMAD2 could partly reverse the proliferation and invasion inhibiting effects of LV-miRNA-200b-3p (Figure 3I–3K). Taken together, these results confirmed that SMAD2 was directly modulated by miR-200b-3p as a target in melanoma cells.

### NEAT1 directly down-regulated miR-200b-3p in melanoma

**Figure 4:**
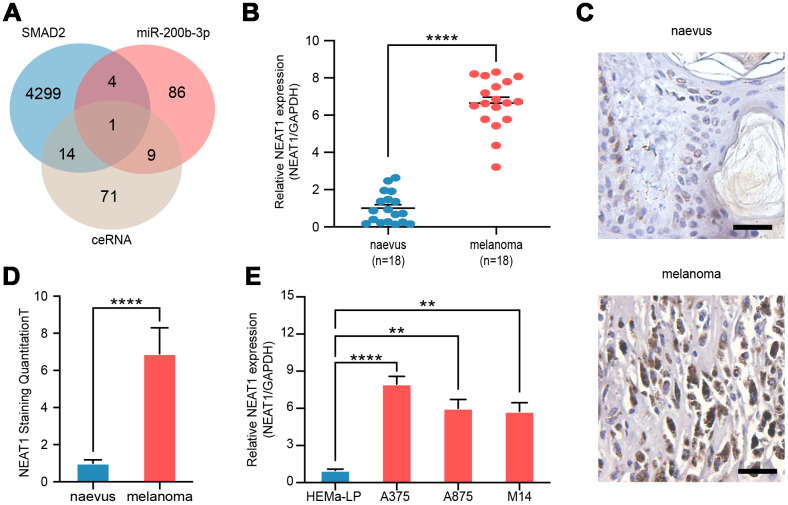
**NEAT1 was upregulated in melanoma cells.** (**A**) Venn diagram of intersection of target lncRNAs predicted by bioinformatics analysis. (**B**) RT-qPCR was performed to measure the level of NEAT1 in benign naevus (n=18) and melanoma tissues (n=18). (**C**) RNAscope detection of NEAT1 expression in melanoma tissues and benign naevus. Scale bars: 100 μm. (**D**) Quantitative analysis of NEAT1 expression in melanoma tissues (n=18) and benign naevus (n=18). (**E**) RT-qPCR was performed to measure the level of NEAT1 in various melanoma cells (A375, A875 and M14) and non-cancer cells, HEMa-LP.

To explore the potential upstream regulation mechanism, we analyzed miR-200b-3p-related lncRNAs, SMAD2-related lncRNAs, and potential lncRNAs in ceRNA regulatory axis and found that the lncRNA NEAT1 was predicted to associate with miR-200b-3p and SMAD2 as a ceRNA (Figure 4A). RT-qPCR and RNAscope assays revealed that expression of NEAT1 in melanoma tissues (n=18) was significantly higher than that in benign naevus (n=18) (Figure 4B–4D). Furthermore, the level of NEAT1 in various melanoma cell lines (A375, A875 and M14) was found to be higher than that of non-cancer cells as well (Figure 4E). These results showed that NEAT1 was up-regulated in melanoma cells.

**Figure 5:**
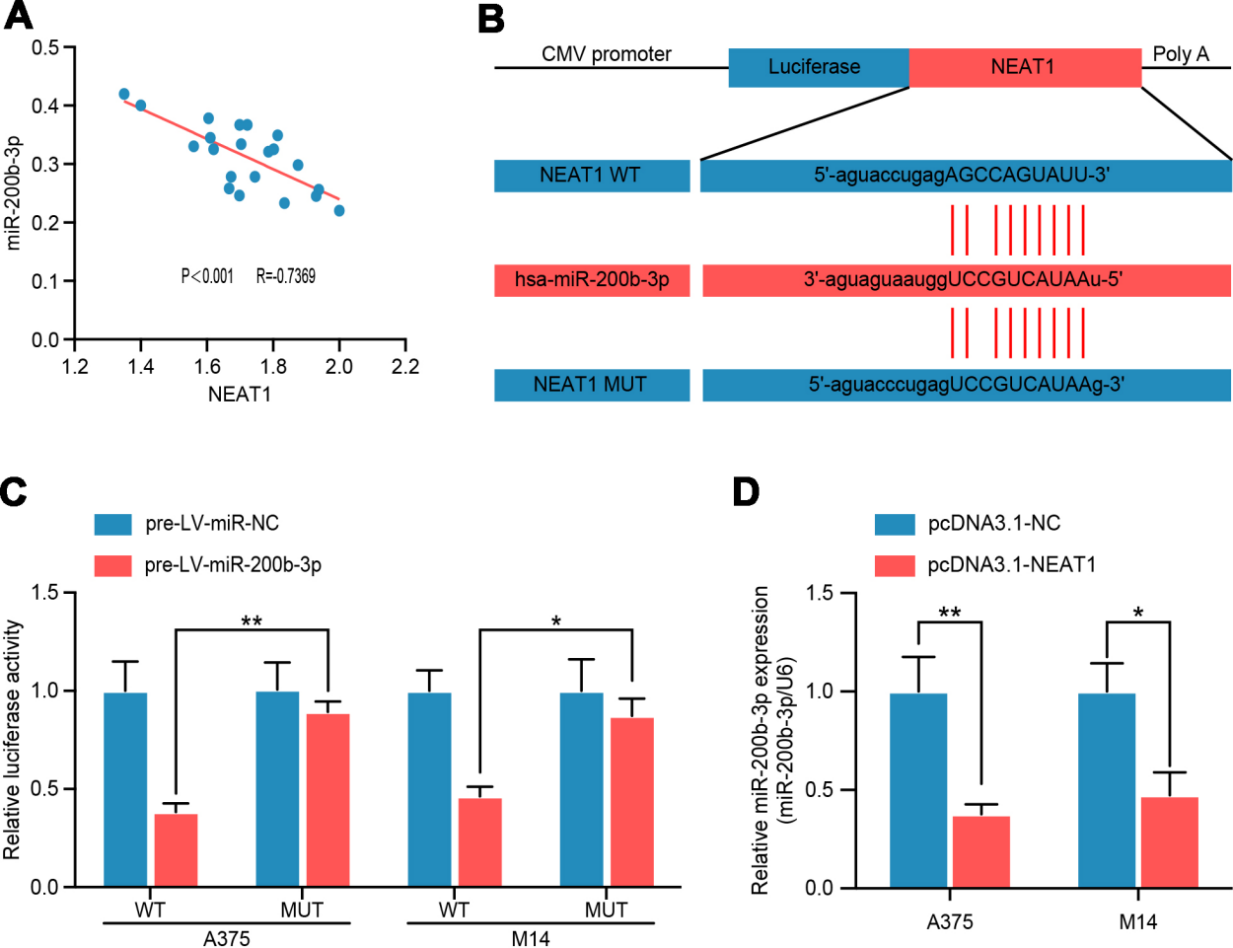
**NEAT1 modulated miR-200b-3p through directly binding.** (**A**) Spearman’s correlation analysis showed the correlation of miRNA-200b-3p and NEAT1 in malignant melanoma tissues(n=18) (R=−0.7369, P < 0.001). (**B**) Schematic view of putative miRNA-200b-3p targeting site in the Wt and MUT 3’-UTR of NEAT1. (**C**) Luciferase reporter assay in A375 and M14 cells transfected with luciferase report plasmids containing NEAT1 3’- UTR (WT or MUT), and pre-miR-control or pre-miR-200b-3p. (**D**) RT-qPCR was performed to measure the level of miR-200b-3p after cells were transfected with pcDNA3.1-NC or pcDNA3.1-NEAT1 in A375 and M14 cells.

To confirm the regulatory relationship between NEAT1 and miR-200b-3p, we analyzed their correlation by Pearson’s analysis and found a negative correlation between NEAT1 and miR-200b-3p in melanoma tissues (R= −0.7369, P<0.001) (Figure 5A). Subsequently, to search for the binding site of miR-200b-3p in the NEAT1 sequence, another multiple sequence comparison analysis was performed (Figure 5B). Luciferase reporter assay demonstrated a considerable decrease in luciferase activity caused by miR-200b-3p in A375 and M14 cells; these inhibitory effects of miR-200b-3p overexpression were abrogated in cells transfected with MUT NEAT1, which reflected the direct binding and endogenous competition (Figure 5C). Moreover, overexpression of NEAT1 decreased miR-200b-3p level in A375 and M14 (Figure 5D). From these results above, we can conclude that NEAT1 suppressed miR-200b-3p as a miRNA sponge in melanoma.

### NEAT1 promoted melanoma proliferation and invasion through inhibiting miR-200b-3p

**Figure 6:**
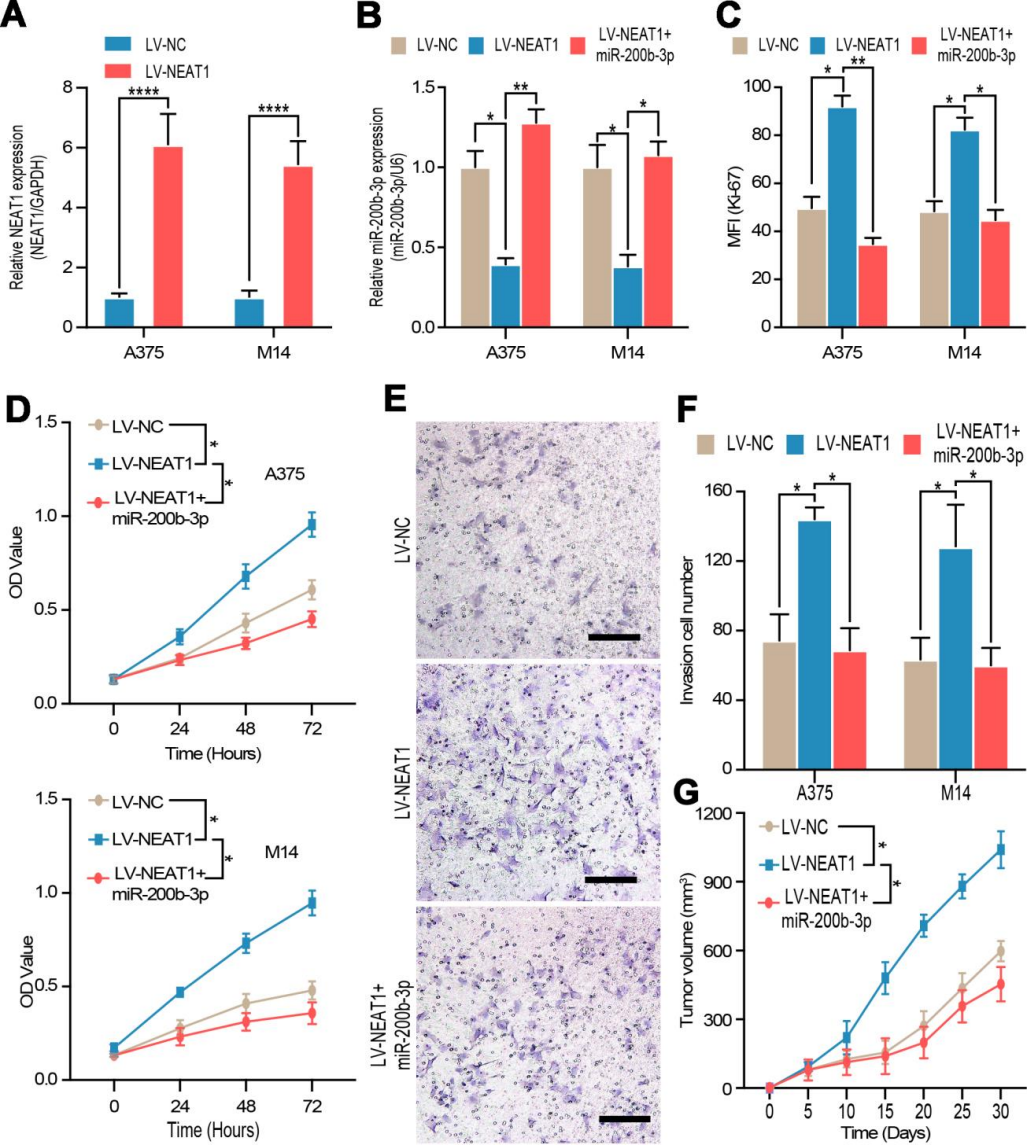
**NEAT1 regulated tumor proliferation and invasion through miR-200b-3p.** (**A**) RT-qPCR was used to test the efficiency of NEAT1 overexpression. (**B**) RT-qPCR was used to detect the expression of miR-200b-3p after cells were transfected with LV-NEAT1 and/or miR-200b-3p mimic. (**C**–**D**) Cell proliferation was measured by flow cytometry and CCK8 after cells were transfected with LV-NEAT1, LV-NEAT1+miR-200b-3p mimic or control (the OD value was measured at 0h, 24h, 48h and 72h). (**E**–**F**) Cell invasion was detected after cells were transfected with LV-NEAT1, LV-NEAT1+ miR-200b-3p mimic or control at 12h. (**G**) Tumor volume was calculated after injection of A375 cells transfected with LV-NEAT1, LV-NEAT1+ miR-200b-3p mimic or control (n=8).

To explore the role of NEAT1 in melanoma, the efficiency of NEAT1 overexpress plasmid was validated by RT-qPCR in A375 and M14 cells (Figure 6A). Then, we discovered that miR-200b-3p mimic could reverse the down-regulation of miR-200b-3p caused by NEAT1 overexpression (Figure 6B), which further confirmed the interaction between NEAT1 and miR-200b-3p. Flow cytometry, CCK-8 assay, and transwell assay were carried out to prove that overexpression of NEAT1 promoted cell proliferation and invasion respectively, while such promoting effects could be blocked by miR-200b-3p mimic (Figure 6C–6F). Lastly, we verified the tumor promoting effects of NEAT1 by transducing overexpression lentivirus, which was abolished by miR-200b-3p mimic *in vivo* (Figure 6G). From these results, it can be deduced that NEAT1 promoted melanoma progression through inhibiting miR-200b-3p.

### NEAT1 up-regulated SMAD2 expression through sponging miR-200b-3p

**Figure 7:**
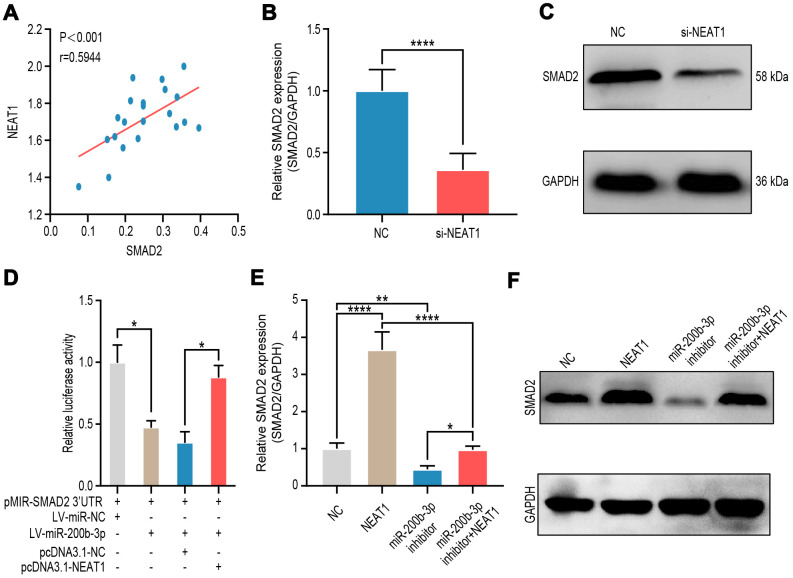
**NEAT1 up-regulated SMAD2 expression through sponging miR-200b-3p.** (**A**) Spearman’s correlation analysis showed the correlation of NEAT1 and SMAD2 in malignant melanoma tissues (n=18) (R=0.5944, P < 0.001). (**B**–**C**) RT-qPCR and western blot were performed to assess mRNA and protein expression of SMAD2 after A375 cells were transfected si-NEAT1 plasmid or NC plasmid treated for 24 hours. (**D**) Luciferase reporter assay in HEK-293T cells transfected with luciferase report plasmids containing SMAD2 3’-UTR (pMIR-SMAD2 3’-UTR) and then were co-transfected with LV-miR-200b-3p or pCDNA3.1-NEAT1. miR-NC and pcDNA3.1-NC were used as control. (**E**–**F**) RT-qPCR and western blot were performed to assess mRNA and protein expression of SMAD2 after A375 cells were transfected with LV-NEAT1 and/or miR-200b-3p inhibitor or control.

Now that the relationships of miR-200b-3p and its upstream regulator NEAT1 as well as its downstream target SMAD2 were clarified, we tended to explore the relationship between NEAT1 and SMAD2. It turned out that NEAT1 was positively correlated with SMAD2 in melanoma tissues (n=18) (Figure 7A). RT-qPCR and western blot presented that NEAT1 down-regulating suppressed the level of SMAD2 (Figure 7B, 7C). In HEK-293T cells, transduction of LV-miR-200b-3p decreased the luciferase activity of SMAD2, however, such effects were rescued by the co-transduction of pcDNA3.1-NEAT1 (Figure 7D). Moreover, RT-qPCR and western blot showed that expression of SMAD2 was upregulated by NEAT1 overexpression, while the miR-200b-3p inhibitor could counteracted these effects regardless of the NEAT1 over-expression (Figure 7E, 7F). The above results provided further evidence that there existed a NEAT1/miR-200b-3p/SMAD2 regulatory axis in melanoma.

### The NEAT1/miR-200b-3p/SMAD2 axis tended to promote melanoma progression by activating EMT pathway and immune responses

**Figure 8:**
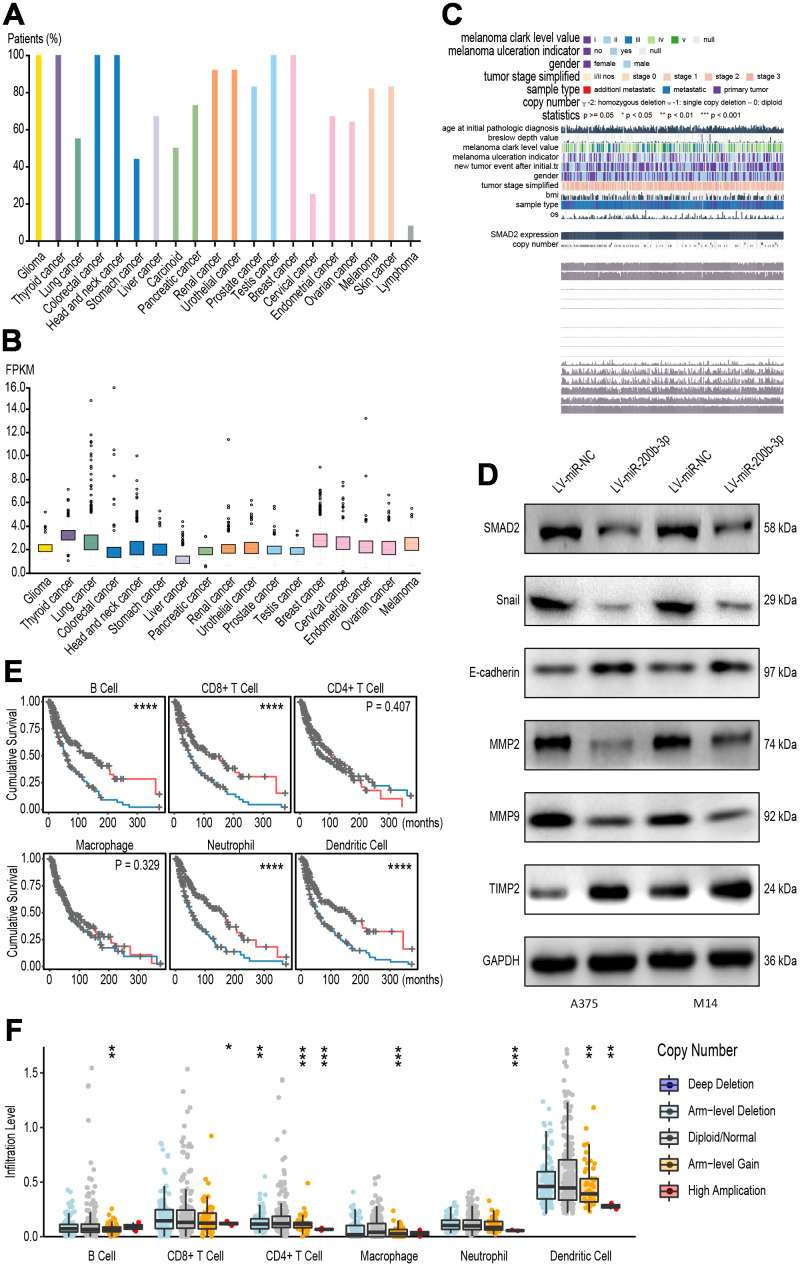
**The NEAT1/miR-200b-3p/SMAD2 axis tended to promote melanoma by immune regulation.** (**A**) Pan-cancer analysis was performed to test the positive rates of SMAD2 in several cancer tissues. (**B**) Pan-cancer analysis showed that SMAD2 was significantly increased In 17 types of cancer tissues. (**C**) Sequencing data and clinical characteristic was analyzed to show SMAD2 related with signaling pathways. (**D**) Western blot determined SMAD2-associated signaling proteins after transfection with LV-miR-200b-3p in A375 and M14 cells. (**E**) Kaplan-Meier plots was drawn to visualize the survival outcomes for different immune infiltrates according to a multivariable Cox proportional hazard model. (**F**) Immune infiltrates assays demonstrated SMAD2-related immune cells after melanoma patients were classified according to SMAD2 levels.

To verify the tumor promoting function of SMAD2 in pan-cancer tissues, we analyzed the positive rates of SMAD2 in several cancer tissues. As shown, most cancers were exhibited as SMAD2 positive, such as colorectal, endometrial, ovarian, renal and thyroid cancers, which indicated that SMAD2 widely expressed in a variety of cancer cells (Figure 8A). In addition, transcription level of SMAD2 was significantly increased in 17 types of cancer tissues, which indicated that SMAD2 had low cancer specificity (Figure 8B). Considering the sequencing data and clinical characteristic, SMAD2 was related with several signaling pathways (Figure 8C). Western blot determined that overexpression of miR-200b-3p in A375 and M14 cells led to the downregulation of SMAD2, and suppressed major SMAD2-associated signaling, including the EMT process(verified by Snail and E-cadherin proteins) and cell invasion (verified by MMP2, MMP9 and TIMP2 proteins) (Figure 8D). To explore the clinical relevance of tumor immune subsets, with the flexibility to correct for multiple covariates in a multivariable Cox proportional hazard model, we drawn Kaplan-Meier plots for immune infiltrates to visualize the survival differences according to TCGA database. The patients were divided by high or low level of immune infiltrates of various types of immune cells. It was illustrated that high infiltrations of B cells, CD8^+^ T cells, neutrophils and dendritic cells were related to better survival outcomes (Figure 8E). Finally, we divided melanoma patients into 5 groups according to SMAD2 levels, immune infiltrates of immune cells were caculated according to SMAD2 levels and the results indicated that different levels of SMAD2 predicted diverse immune infiltration patterns, indicative of the correlation between SMAD2 and immune infiltrates (Figure 8F). In brief, it displayed that the NEAT1/miR-200b-3p/SMAD2 axis may promote melanoma by activating EMT and immune responses.

## DISCUSSION

Melanoma is a genetically heterogeneous malignancy with high mutation frequency, and is initiated by interactions of genes and environmental risk factors, ultraviolet radiation (UVR) in particular [23]. However, in Asian patients, melanoma develops from nevus located in body parts which are not much exposed to ultraviolet [24]. This discrimination further illustrates that the pathogenesis of melanoma demands to be discovered. Novel genes and signaling pathways in melanoma tumorigenesis and progression are yet to be identified. Existing researches have recognized the critical roles of ncRNAs in various pathophysiological processes. ncRNAs, including miRNAs and lncRNAs, which are frequently dys-regulated in a variety of cancer and have been reported to exert influences on various cell processes, such as transcription, chromosome remodeling, intracellular trafficking [25]. In a sense, these ncRNAs act as either oncogenes or tumor-suppressor genes by regulating tumor-related genes or pathways [26].

Dys-regulation of miR-200b-3p has been reported to associate with the development and progression of some cancers. For one thing, miR-200b-3p functions as an oncogene and promote the proliferation and invasion of cancers, and is found to be significantly up-regulated in various cancers such as lung cancer [27], oral squamous cell carcinoma [28], pancreatic cancer [29] and prostate cancer [30]. For another, miR-200b-3p exhibits tumor suppressive effects through various pathways in breast cancer [31], glioblastoma [32], and colorectal cancer [33]. Hence, it could be conceivably hypothesized that the cancer-specific miR-200b-3p exerts cancer-promoting or anti-cancer effects in a cancer type-dependent manner. In our study, miR-200b-3p was detected to be down-regulated in melanoma, and high level of miR-200b-3p in melanoma patients fostered a poor survival outcome. Overexpression of miR-200b-3p was found to suppress the proliferation and invasion of melanoma cells. These findings indicated that downregulation of miR-200b-3p promoted tumorigenesis and development of melanoma, thus might serve as a potential biomarker for the prognosis of patients with melanoma.

SMAD2, which was verified to be a direct functional target of miR-200b-3p, is characterized as a canonical downstream regulator of TGF-β signaling and involved in several cell process, such as cell cycle, cell growth, cellular fibrosis, EMT, etc [34]. In our study, SMAD2 was found up-regulated in melanoma tissues and cell lines. Not surprisingly, the correlation between NEAT1 and SMAD2 was proved to be positive. Overexpression of SMAD2 could partly reverse the proliferation and invasion inhibiting effects by miRNA-200b-3p overexpression, thus reflecting the tumor promoting effects of SMAD2. Furthermore, pan-cancer analysis revealed that an enhanced expression of SMAD2 was detected in most cancers, and the SMAD2 related signaling pathways were activated as well. By means of western blot, we validated that overexpression of miR-200b-3p resulted in the suppression of SMAD2 signaling, thus weakening the EMT and cell invasion process of melanoma cells. Hence, we can deduce that the tumor promoting effects of the NEAT1/ miR-200b-3p/ SMAD2 signaling pathway in melanoma is partly attributed to the activation of EMT.

Multiple studies have elaborated that in most cancer types, levels of NEAT1 seem to be elevated, and its tumor promoting effects are accepted as well [35]. By competitively interacting with miRNA response elements, lncRNAs may act as ceRNAs or miRNA sponges so as to abolish miRNA target suppression [36]. Therefore, the function of NEAT1 as a miRNA sponge is highlighted. Few research has established the role of NEAT1 in melanoma progression, and some miRNAs are identified in the meantime, such as miR-23a-3p, miR-495-3p, and miR-224-5p [37–39]. In accordance with previous studies, our work verified that NEAT1 was highly expressed in melanoma. Moreover, we proved it *in vitro* and *in vivo* that NEAT1 regulated the proliferation and invasion of melanoma cells as a sponge by directly binding miR-200b-3p. Nevertheless, despite the definite regulatory connections between NEAT1 and miR-200b-3p, there exists a controversial issue. According to the mechanism of miRNA processing, it is of common acceptance that miRNA sponging happens in the cytoplasm [40]. However, the results of RNAscope in Figure 4B and the consistent descriptions from previous literature [41] demonstrated that NEAT1 was localized mainly in the nucleus, which is contradictory to the miRNA sponge role of NEAT1. Therefore, we hypothesize that NEAT1 was transported to the cytoplasm to accomplish its role as a ceRNA. Meanwhile, further research should be undertaken to investigate the underlying mechanism of this transportation process.

In normal skin, by expressing a cell-cell adhesion molecule E-cadherin, keratinocytes can prevent melanocytes from escaping the epidermis [42]. However, in melanoma, such adhesion molecules are no longer expressed, so that tumor cells evade the epidermal layer, indicative of their loss of epidermal properties. As a result, melanoma cells achieve mesenchymal transition which promotes tumor invasiveness and progression [43]. As a critical mechanism of cancer progression and metastasis, the EMT process involves various pathways, which includes PI3K/AKT/mTOR, RAS/RAF/MEK/ERK, Wnt/β-catenin, and Transforming Growth Factor-β (TGF-β) [44]. Here, we corroborated that overexpression of miR-200b-3p hampered the EMT of melanoma cells by upregulating E-cadherin and downregulating Snail, thus enhancing cell-cell adhesion and dampening cell invasiveness and metastasis. Now that previous literature has illustrated the participation of miR-200 family in EMT and SMAD2 is regarded as the subsequent effector of the TGF-β signaling pathway in EMT, it’s reasonable to infer that the NEAT1/miR-200b-3p/SMAD2 axis can regulate the EMT process of melanoma cells.

It has been widely acknowledged that tumor cells are antigenic, and the immune system can be activated to attack against tumor spontaneously [45]. As one of the most immunogenic tumors, melanoma is prone to respond favorably to immunotherapy [46]. There has been growing interests in the crosstalk between melanoma and immune cells in the tumor microenvironment. In line with recent study that the existence of immune infiltrates in tumor deposits is a good omen for melanoma [47], our work demonstrated that high infiltrating rates of B cells, CD8^+^ T cells, neutrophils and dendritic cells in melanoma deposits predicted favorable overall survival outcomes. In our study, melanoma patients were divided into 5 groups according to varying SMAD2 levels, and then we discovered several immune cells were related to SMAD2 through the immune infiltrates assay. For instance, high amplification of SMAD2 was related to low infiltrates levels of dendritic cells, macrophages and CD8^+^ T cells, thus forming an immune-suppressive microenvironment and making ways for the immune escape of melanoma cells. Coincidently, high level of SMAD2 was associated with a poor prognosis simultaneously, indicating that SMAD2 may serve as a novel prognostic marker for melanoma along with miR-200b-3p. Collectively, we have good reasons to believe that NEAT1/miR-200b-3p/SMAD2 signaling pathway may promote melanoma partly by immune regulation.

In conclusion, our study demonstrated the role of miR-200b-3p which is sponged by NEAT1 and functions via SMAD2. The NEAT1/miR-200b-3p/SMAD2 signaling pathway promotes the proliferation and invasion of melanoma cells by activation of EMT. Meanwhile, this regulatory axis is related with immune responses in melanoma. Our work provides novel molecular mechanisms and potential therapeutic targets for melanoma.

## MATERIALS AND METHODS

### Clinic samples collection

Before the study, signed written informed consents were obtained from all the enrolled participants. 18 melanoma tissues and 18 benign nevus tissues were collected from patients who received wide local excision surgery and nevus resection respectively, from May 2018 to August 2019. All tissues were stored in liquid nitrogen until use. This research received the approval of the Ethics Committee of Changhai Hospital, Naval Medical University.

### Cell culture

HEMa-LP, A375, A875, M14, HEK-293T cells were obtained from the Cell Bank of the Chinese Academy of Sciences. All the cells were cultured in Dulbecco's modified Eagle medium (Invitrogen, Carlsbad, California, USA) with 10% FBS (Gibco BRL, Gaithersburg, MD) at 37°C with 5% CO_2_.

### Cell viability assay

Cell viability was detected by Cell Counting Kit-8 (CCK-8) reagent (Dojindo, Tokyo, Japan) according to the manufacturer’s instructions. All the experiments were conducted at least in triplicate.

### Cell transfection

The GFP-labeled lentivirus vectors containing the miR-200b-3p mimic lentivirus (LV-miR-200b-3p) and the corresponding control miRNA lentivirus (LV-miR-NC), the NEAT1 overexpress lentivirus (LV-NEAT1), and the corresponding control lentivirus (LV-NC), as well as the small interfering RNA si-NEAT1 and the corresponding control si-NC were obtained from GeneChem (Shanghai, China). Transduction with the lentiviral vectors was conducted using transduction reagents and 8 mg/ml Polybrene (GeneChem) for 12 h. The siRNA transduction was conducted with Lipofectamine3000 Reagent according to the manufacturer's instructions (Invitrogen, Carlsbad, California, USA). Stable cell lines were then established, and the efficiency was confirmed by RT-qPCR.

### Flow cytometry

Melanoma cells were collected and 1 ml of 1× FOXP3 Fix/Perm solution (BioLegend, Inc., San Diego, CA, USA) was added to each for 20 min and then were stained with anti-Ki67 antibodies (1:20; cat. no. 350514; BioLegend, Inc.) for 30 min according to the manufacturer's instructions. Isotype IgG antibody (1:20; cat. no. 400141; BioLegend, Inc.) was used as a control. Treated samples were analyzed with a CytoFLEX cytometer and CytExpert software (version 2.0) (both from Beckman Coulter Life Sciences, College Park, MD, USA). The mean fluorescence intensity (MFI) of Ki67 was detected. MFI refers to the mean of the fluorescence intensity in the fluorescence channel. The experiments were performed in triplicate.

### Luciferase reporter assay

A375 and M14 cells were seeded in a 24-well plate, and then were co-transfected with luciferase report plasmids containing wild type (WT) or mutant (MUT) SMAD2 3’- UTR, and LV-miRNA-200b-3p or LV-miRNA-NC to test the binding between miR-200b-3p and SMAD2; the luciferase reporter plasmids containing WT or MUT NEAT1 coding regions was co-transfected to A375 and M14 cells with pre-miR-200b-3p or an empty plasmid vector pre-miR-NC to test the binding of NEAT1 and miR-200b-30. The relative dual-luciferase activity of cell lysates was normalized to *Renilla* luciferase activity and detected with a Dual-Luciferase Reporter Assay system (Promega Corp., Madison, WI, USA). The experiments were performed in triplicate.

### RNAscope

The RNAscope probe targeting NEAT1 was designed and synthesized by Advanced Cell Diagnostics. The RNAscope 2.5 High Definition (HD)-BROWN Assay kit was used to evaluate NEAT1 expression according to the manufacturer’s instructions (Advanced Cell Diagnostics, Newark, CA, USA).

### Reverse transcription-quantitative PCR (RT-qPCR)

Total RNA was extracted by TRIzol (Invitrogen, Carlsbad, California, USA) from tissues and cells, and then was quantified by a NanoDrop spectrophotometer (NanoDrop Technologies; Thermo Fisher Scientific, Inc.). The PrimeScript™ RT Reagent Kit (TaKaRa Biotechnology, Co., Ltd., Dalian, China) was used to reversely transcribe total RNA to cDNA. Next, SYBR Green PCR Master Mix (TaKaRa Biotechnology) was used to perform RT-qPCR. NEAT1 and SMAD2 mRNA expressions were normalized to GAPDH expression. The miScript Reverse Transcription Kit (Qiagen GmbH, Hilden, Germany) and miScript SYBR Green PCR Kit (Qiagen GmbH) were used to assess miR-200b-3p level for reverse transcription and RT-qPCR respectively. U6 acted as the endogenous control. All reactions were processed on the Applied Biosystems 7500 Real-Time PCR System (Thermo Fisher Scientific, Inc.). Relative gene expression was analyzed using the 2^−ΔΔCt^ method.

### Transwell assay

All the transwell chambers (Corning Incorporated, Corning, NY, USA) were coated with 50 μL Matrigel (354480; BD Biosciences) and incubated at 37°C for 1 h in advance. Next, 5 × 10^4^ cells re-suspended in 200 μL FBS-free culture medium were seeded in the upper chamber. The bottom chamber was filled with 500 μL culture medium containing 20% FBS. After 24 h of incubation, a cotton swab was used to eliminate noninvasive cells in the upper chamber. Invasive cells were fixed in 4% paraformaldehyde, stained with 0.5% crystal violet, and imaged under an inverted microscope. Finally, invasive cells in five randomly selected fields were counted.

### Western blot

Treated cells were lysed in RIPA buffer supplemented with phenylmethylsulphonyl fluoride (Beyotime Institute of Biotechnology, Haimen, China). The BCA method was used to assess protein concentration. Proteins were separated by 12.5% SDS-PAGE and transferred to PVDF membrane and immunoblotted with the following antibodies: anti-SMAD2 (1:1000, ab33875, Abcam, Cambridge, MA, USA), anti-Snail (1:1000, Abcam, ab53519), anti-E-cadherin 1:1000, (1:1000, Abcam, ab231303), anti-MMP-2 (1:1000, Abcam, ab97779), anti-MMP9 (1:1000, Abcam, ab137867), anti-TIMP2 (Abcam, ab180630), and anti-GAPDH (1:1000, Abcam, ab181603). After rinsed with TBST, the membranes were incubated with HRP-conjugated secondary antibody (1:1,000; cat. no. A0208; Beyotime Institute of Biotechnology), developed with an ehanced chemiluminescence regent (GE Healthcare Bio-Sciences, Pittsburgh, PA, USA) and visualized by Image Lab software (Bio-Rad Laboratories, Hercules, CA, USA). The experiments were performed in triplicate.

### Xenograft mouse model

A375 cells (1x10^7^) stably expressing LV-miR-NC, LV-NC, LV-miR-200b-3p, LV-NEAT1, LV-miR-200b-3p+LV-NEAT1 were subcutaneously injected into the left flank area of 4-week-old nude mice (n=8 mice/group). Tumor volumes were determined every 5 days (0.5×length×width^2^). Five weeks later, the mice were sacrificed and xenografts were assessed and weighed. Experiments on animals were approved by the Ethics Committee for Animal Experimentation of the Ruijin Hospital, Shanghai Jiao Tong University School of Medicine and strictly conformed to the Institutional Guidelines for Use and Care of Laboratory Animals.

### Silico analysis

In order to identify genes that may be involved in melanoma, we analyzed differential miRNAs according to several GEO datasets (GSE35579, GSE143231, GSE143777, GSE138710, GSE138412 and GSE117666). To identify the possible target gene of miR-200b-3p in melanoma, bioinformatics analysis was conducted using several bioinformatics databases including PITA (http://genie.weizmann.ac.il/pubs/mir07/mir07_data.html), microT (https://bio.tools/DIANA-microT), TargetScan (http://www.targetscan.org/vert_72/) and RNA sequencing results of GSE149941. The survival time and immune infiltration were analyzed according to TCGA database.

### Statistical analysis

All data were processed as the mean ± SEM. ANOVA, Student's t-test and chi-square test were conducted with SPSS 21.0 software (IBM Corp., Armonk, NY, USA). Tukey's test was used to test all pairs of columns. Log-rank test was performed and Kaplan-Meier survival curves were plotted. The P-values were two-sided and a value of <0.05 was regarded as statistically significant.
